# Poly(3,4-ethylenedioxythiophene) Electrosynthesis in the Presence of Mixtures of Flexible-Chain and Rigid-Chain Polyelectrolytes

**DOI:** 10.3390/polym13223866

**Published:** 2021-11-09

**Authors:** Varvara Kabanova, Oxana Gribkova, Alexander Nekrasov

**Affiliations:** A.N. Frumkin Institute of Physical Chemistry and Electrochemistry RAS, Leninskii prospect 31, 119071 Moscow, Russia; kabanovavar@gmail.com (V.K.); oxgribkova@gmail.com (O.G.)

**Keywords:** PEDOT, electropolymerization, polyelectrolyte, ammonia sensor, spectroelectrochemistry

## Abstract

The electrochemical synthesis of poly(3,4-ethylenedioxythiophene) (PEDOT) was first carried out in the presence of mixtures of flexible-chain and rigid-chain polyacids and their Na-salts. Earlier on with the example of polyaniline, we have shown the non-additive effect of the rigid-chain component of polyacid mixtures on the electrodeposition of polyaniline films, their morphology and spectroelectrochemical properties. In this study, we confirmed the non-additive effect and showed that such mixed PEDOT–polyelectrolyte films possess unique morphology, spectroelectrochemical and ammonia sensing properties. The electrosynthesis was carried out in potential cycling, galvanostatic and potentiostatic regimes and monitored by in situ UV–Vis spectroscopy. UV–Vis spectroelectrochemistry of the obtained PEDOT–polyelectrolyte films revealed the dominating influence of the rigid-chain polyacid on the electronic structure of the mixed complexes. The mixed PEDOT–polyacid films demonstrated the best ammonia sensing performance (in the range of 5 to 25 ppm) as compared to the films of individual PEDOT–polyelectrolyte films.

## 1. Introduction

Conductive polymers (CPs) such as poly(3,4-ethylenedioxythiophene) (PEDOT), polyaniline (PANI) and polypyrrole reveal a unique combination of physicochemical, electrochemical, optical and magnetic characteristics that make them promising for use as antistatic or conductive coatings, components of electrochromic, electroluminescent and organic photovoltaic devices, supercapacitors, chemical and biological sensors [1]. This is the main reason for the intensive research and development of such materials.

Besides the change in conductivity, the doping of a CP leads to a change in the electronic structure of the CP that is accompanied by a change in the optical properties in the UV, visible and near-IR (UV–Vis–NIR) regions. This allows them to be used in electrochromic displays and optical sensors for gaseous or liquid substances [2,3].

A special place among CPs is occupied by PEDOT. This is due to its properties, including optical transparency in the conducting state, sufficiently high conductivity, high stability and ease of synthesis [4,5].

The synthesis of PEDOT can be carried out both by chemical and electrochemical methods in the presence of polyelectrolytes (PEs). They work as counterions for PEDOT chains and stabilize the insoluble charged PEDOT in aqueous dispersion. There are a number of PEs used for chemical EDOT polymerization for improving its solubility and modifying the PEDOT properties [4,6,7,8,9]. The most popular PE is polystyrenesulfonate (PSS), which forms with PEDOT a blend best known as PEDOT:PSS. An enormous number of studies are devoted to the investigation of its morphological, structural, electrical and optical properties, along with its applications in various devices [10].

The use of PEs of different structures in the electrochemical synthesis of PEDOT makes it possible to modify its physicochemical properties and the morphology of the films obtained [11,12,13,14,15]. The electrochemical method of PEDOT synthesis has a number of significant advantages, such as one-step formation of an oxidant-free homogeneous film with controlled thickness, morphology and good adhesion to the substrate.

One of the most promising applications of PEDOT films is the possibility of using them for the detection of gases. Among them, ammonia is a dangerous environmental pollutant, usually formed as a result of anthropogenous processes and the operation of industrial facilities. It is a toxic, flammable, colorless gas that can damage the cells of the human body, causing damage to the skin, eyes and respiratory tract. It is used as a refrigerant in industrial refrigeration units, in dairies, meat processing plants and other facilities, which are often located directly on the territory of settlements. Therefore, the control of the concentration of ammonia in air is an important and urgent task. The UK Health and Safety Executive (HSE) has set the long-term exposure limit in the air (8 h TWA reference period) for ammonia at 25 ppm [16].

In the majority of ammonia sensors based on CP films, two popular sensing techniques are used: chemiresistive and optical methods [3,17,18,19,20]. The use of the optical detection method has a number of significant advantages, such as reduced influence of humidity on the operation of the detector, insensitivity to electromagnetic and radiation fields and remote control—the ability to transmit an analytical signal without distortion over long distances [3,18,19,21]. In comparison to most sensors based on metal oxides, sensors based on CPs have good mechanical properties and stability in air, as well as sensitivity at room temperature [3,17,18,19].

The lowest detection limits of optical sensors based on CPs were 12 ppm for dye-doped PPy [22], 2.73 ppm for PEDOT [21] and 1–5 ppm for PANI [23,24]. PANI is more popular among CP sensing layers due to the very high rate and amplitude of the optical response on ammonia action [23,25]. In the studies [25,26], it was shown that PANI/PEDOT bilayer structures have a higher sensitivity to ammonia due to their wider spectral region than for individual PANI. The authors in [27] report that the detection limit of the bilayer PANI/PEDOT was 7.86 ppm with a response time of 2.33 min.

At the moment, the sensing properties of PEDOT films in relation to ammonia have been insufficiently studied [21,27,28,29,30,31,32], despite their high sensitivity to the effect of a reducing gas (a wide range of changes in resistance and optical density at a certain wavelength). Most often, optical sensors based on the films cast from commercially available chemically synthesized water dispersion of PEDOT:PSS are used [28,29,30,31]. For the best optical sensor based on PEDOT, the detection limit was 2.73 ppm, response amplitude—9.03%, response time—1.19 min and recovery time—5.47 min [21].

Earlier [33], the electrosynthesis of PANI was performed in the presence of a mixture of polyacids with different flexibilities of the polymer chain. It was shown that the rigid-chain polyacid has a dominating influence on the synthesis and properties of the resulting PANI complex. The possibility of combining the specific electronic and spectroelectrochemical properties of PANI complexes with polyacids of various chain flexibilities in a single film is the advantage of such a synthesis. In this work, we have tried a similar approach for EDOT electropolymerization. The electrochemical synthesis of PEDOT in the presence of mixtures of sulfonated PEs of various structures and flexibilities of the polymer chain was carried out for the first time. The features of EDOT electropolymerization in the presence of flexible-chain and rigid-chain PEs and their mixtures in acid and salt forms were studied by in situ spectroelectrochemistry in UV–Vis regions. The resulting films were characterized by UV–Vis–NIR spectroscopy and UV–Vis spectroelectrochemistry, as well as atomic force microscopy. The ammonia sensing properties of the obtained PEDOT films were investigated.

## 2. Materials and Methods

**Figure 1:**
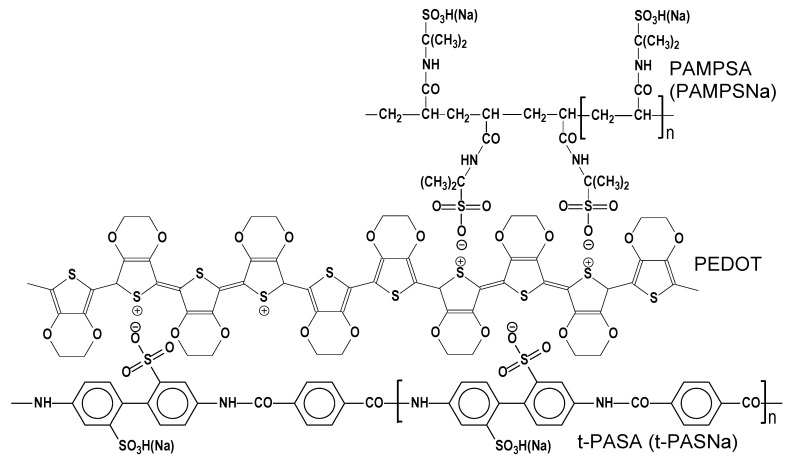
Structures of monomer units of polyelectrolytes used in this study (PAMPSA(Na) and t-PASA(Na)) and simplified scheme of their electrostatic interaction with charged PEDOT moieties (scheme of bipolaron is based on [35]).

Electrochemical polymerization of 3,4-ethylenedioxythiophene (EDOT) was carried out in the presence of the following water-soluble PEs (Figure 1): flexible-chain poly-(2-acrylamido-2-methyl-1-propanesulfonic acid) (PAMPSA), rigid-chain poly-(4,4′-(2,2′-disulfonic acid)-diphenylene-tere-phthalamide) (t-PASA), their mixtures and their sodium salts. PAMPSA (Sigma-Aldrich Co., St. Louis, MO, USA, *M*_w_ = 2,000,000, 15% aqueous solution) was converted to Na^+^ form during the pH-titration process. The laboratory-synthesized Na^+^ salt of t-PASA [34] with *M*_w_ = 40,000 was converted to the H^+^ form using an ion exchange column. All used PEs were purified from low molecular weight fractions by dialysis against distilled water (dialysis membrane ZelluTrans MWCO 8000–, Carl Roth GmbH & Co. KG, Karlsruhe, Germany). Before the synthesis, EDOT (Sigma-Aldrich Co., St. Louis, MO, USA) was distilled under vacuum (20 mmHg, 125 °C).

The concentration of EDOT for all solutions was 0.01 M. The ratio of concentrations of EDOT to sulfonic groups was always kept as 0.5 mol/g-equivalent of sulfonic groups. The concentrations of PEs were calculated taking into account their basicity [33]. The compositions of PAMPSA/t-PASA mixtures were 2:1, 1:1 and PAMPSNa/t-PASNa 1:1 with respect to sulfonic groups. Aqueous solutions of polyacids and their salts of the required concentration were prepared one day before the synthesis of PEDOT. In order to obtain a homogeneous mixture of poorly soluble EDOT in water, the solutions were intensively stirred for 2 h with a heating to ~60 °C.

EDOT polymerization was carried out in three regimes: potential cycling (PC) (−0.6–1.0 V, potential sweep rate 50 mV/s), galvanostatic (GS) (0.05 mA/cm^2^) and potentiostatic (PS) (0.9 V) on purified glass electrodes coated with a transparent conductive layer of SnO_2_:F (FTO), with a surface resistance of ~7 Ω/square. The working surface of the electrode was 2 cm^2^. As a counter electrode, a platinum foil was used. The reference electrode was a silver–silver chloride electrode in saturated KCl. The polymerization was carried out in a specially designed three-electrode spectroelectrochemical quartz cell. The electrochemical parameters of the synthesis were controlled and recorded using a HA-501G potentiostat/galvanostat (Hokuto Denko Ltd., Meguro-ku, Japan) and a Nicolet 2090 digital storage oscilloscope (Nicolet Test Instruments Division, Madison, WI, USA). Simultaneously, the electronic UV–Vis absorption spectra (380–900 nm) were registered every 2 s using a high-speed scanning single-beam spectrophotometer Avantes 2048 (Avantes, Apeldoorn, The Netherlands). The electrodeposition of PEDOT films was carried out until a charge of 50 mC/cm^2^ was reached. Electrochemical and spectroelectrochemical studies of the PEDOT films thus produced were taken with the same equipment in 0.5 M NaClO_4_ aqueous solution.

Spectroscopic studies of the dry PEDOT–PE films on air in the UV–Vis–NIR spectral area (350–1850 nm) were performed using a UV3101PC spectrophotometer (Shimadzu Scientific Instruments Inc., Columbia, MD, USA).

Atomic force microscopy (AFM) of PEDOT films obtained in GS mode was performed on an Enviroscope AFM microscope with a Nanoscope V controller (Bruker GmbH, Berlin, Germany) in tapping mode. The thickness of the films depending on PEs used was 200–300 nm and was measured by the MII-4 microinterferometer (LOMO, St. Petersburg, Russia).

A comparative study of the sensing properties of the PEDOT–PE films obtained in GS mode with respect to 5 and 25 ppm of ammonia was carried out using optical detection. The films on transparent FTO substrates were placed in a closed 5 cm quartz cuvette filled with ammonia vapors over 5 mm layer of the aqueous solutions of different NH_3_ concentrations prepared by dilution of the 10% solution. The concentrations of ammonia in air were calculated using interpolated calibration curve based on the values of NH_3_ gas partial pressure over ammonia aqueous solutions tabulated in [36].

The spectral changes of the films under ammonia vapor in the UV–Vis regions were recorded by Avantes 2048 spectrophotometer every 2 s.

The response amplitude was calculated as a relative absorbance change (∆A) at wavelengths specific for each film. The response time (t_r_) was the time required to reach 90% of ∆A.

(1) $$\Delta A=\frac{A_{{\mathrm{NH}}_{3}\mathrm{vap}}-A_{\mathrm{air}}}{A_{\mathrm{air}}}\cdot 100\%,$$

where A_NH3vap_ is the value of absorbance when the sample is exposed to NH_3_, and A_air_ is the value of absorbance when the sample is exposed to air.

## 3. Results and Discussion

### 3.1. Electrochemical Synthesis of PEDOT

#### 3.1.1. Electrochemical Data

**Figure 2:**
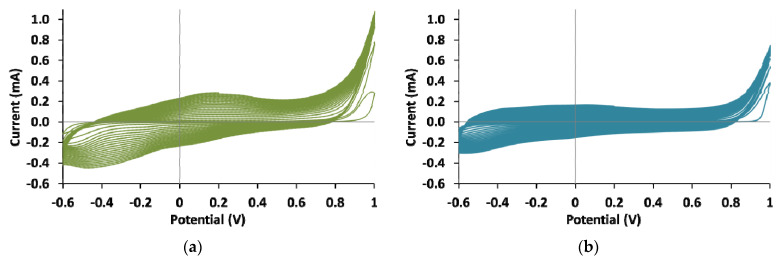
The cyclic voltammograms obtained during the electrosynthesis of PEDOT in the regime of cycling potential in the presence of the mixtures of PAMPSA/t-PASA (1:1) (**a**) and PAMPSNa/t-PASNa (1:1) (**b**).

The CV curves obtained during PEDOT electrosynthesis in the PC regime are shown in Figure 2. The CV curve of PEDOT film with polyacid mixture shows a shape similar to the curve obtained during the synthesis in the presence of t-PASA [37]—the maximum current in the region of 0.1–0.3 V is more pronounced than in the presence of flexible-chain polyacid [38] and is shifted in the anodic direction by 60–70 mV (Figure 2a). In the case of EDOT polymerization in the presence of PE mixture in salt form (Figure 2b), one can see a curve similar in shape to the curves registered during the electrosynthesis of PEDOT in aqueous medium [39,40] and synthesis in the presence of PAMPSA, PAMPSNa and t-PASNa (the absence of clearly visible peaks on CVs) [37,38].

Table 1 shows the parameters of PEDOT electrosynthesis in various regimes in the presence of all selected PEs. It can be seen that the onset potential of monomer oxidation (E_PC_), determined from CV, in the case of polyacids and their mixtures is lower than this one for the synthesis in the presence of salt forms of PEs. In the case of mixtures of polyacids, the lowest E_PC_ was observed.

**Figure 3:**
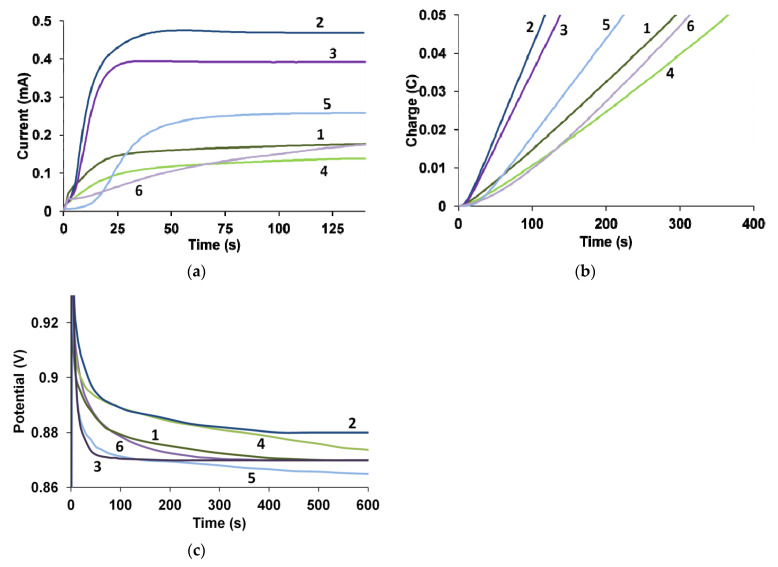
Kinetic curves of current (**a**) and charge (**b**) obtained during PS synthesis and potential (**c**) obtained during GS synthesis in presence of PAMPSA (1), t-PASA (2), PAMPSA/t-PASA (1:1) (3), PAMPSNa (4), t-PASNa (5), PAMPSNa/t-PASNa (1:1) (6).

The greatest differences are observed in the case of synthesis in the PS regime (Figure 3a,b). The synthesis of PEDOT in the presence of PAMPSA and its salt proceeds at the lowest current (I_PS_). The highest synthesis currents are observed in the presence of t-PASA and the mixture of polyacids. The duration of the induction periods (T_PS_) of EDOT polymerization in the presence of various PEs was calculated based on the kinetic curves of charge (Figure 3b) during the synthesis. It can be seen in Table 1 that the synthesis of PEDOT in the presence of PEs in the salt form is characterized by a slightly longer induction period than that for the synthesis in the presence of acid forms of PEs.

Summarizing, one can see that the synthesis in the presence of t-PASA and the mixtures of PAMPSA/t-PASA proceeds much more easily (higher current and shorter induction period) than in the presence of PEs in the salt form and PAMPSA. It was shown [11] that during EDOT electropolymerization in the presence of rigid-chain polyacids, the formation of radical cations dominated. Since radical cations are the driving force of EDOT electropolymerization, this leads to a higher rate and current of PS synthesis.

In the case of GS synthesis, close values of potential (E_GS_) of EDOT electropolymerization were observed (Figure 3c, Table 1).

#### 3.1.2. Spectroelectrochemical Data

**Figure 4:**
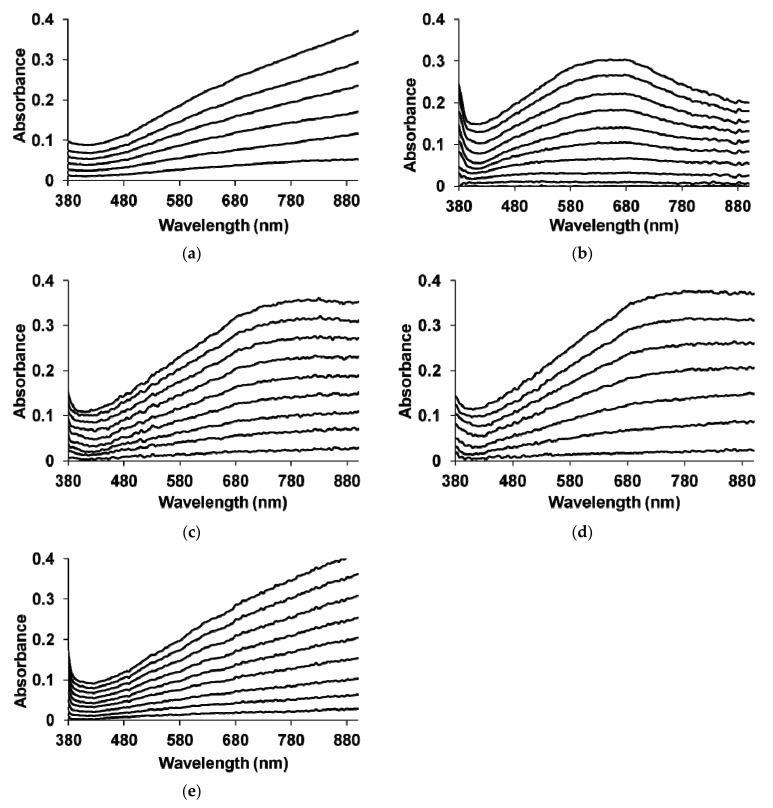
Electronic UV–Vis absorption spectra of PEDOT films formed on the working electrode during GS synthesis in aqueous solutions of PAMPSA (**a**), t-PASA (**b**), PAMPSA/t-PASA (1:1) (**c**), PAMPSA/t-PASA (2:1) (**d**) and PAMPSNa/t-PASNa (**e**).

The growth of the optical absorption spectra in UV–Vis region during the polymerization of EDOT in the GS regime is shown in Figure 4.

In the process of PEDOT synthesis in the presence of PAMPSA, an intense increase in the absorption in the region of wavelengths longer than 600 nm is observed (Figure 4a), which extends to the NIR region of the spectrum indicating the formation of the highly conductive second oxidized form (bipolarons) [40,41,42,43]. During EDOT electropolymerization in the presence of PAMPSNa and t-PASNa, as well as their mixture (Figure 4e), the evolution of absorption spectra is similar to that for the synthesis in the presence of PAMPSA (Figure 4a). Thus, in the case of EDOT electropolymerization in the presence of salt forms of PEs and their mixture, the chemical structure of PE molecules does not affect the course of PEDOT synthesis and electronic structure of the growing film.

Investigating the nature of the difference in the absorption spectra during the polymerization of EDOT in the presence of t-PASA (Figure 4b), one can observe the formation of a wide absorption maximum located at about 600–700 nm. In this case, the increase in absorption in the NIR region is less pronounced than in PAMPSA (Figure 4a). In the case of synthesis in the presence of the PAMPSA/t-PASA mixture (Figure 4c,d), one can see the broad absorption maximum near 680–780 nm, which is likely caused by conjugation breaks in the PEDOT chains as in the case of synthesis in t-PASA. A pronounced absorption plateau in the NIR region (Figure 4c,d) indicates the formation of bipolarons occurring to a greater extent compared to the synthesis in t-PASA. Thus, the presence of rigid-chain polyacid in PAMPSA/t-PASA mixtures produces an obvious influence on the electronic structure of electrodeposited PEDOT complexes even at twice higher content of the flexible-chain polyacid.

### 3.2. Characterisastion of PEDOT–PE Films

#### 3.2.1. UV–Vis–NIR Absorption Spectroscopy

**Figure 5:**
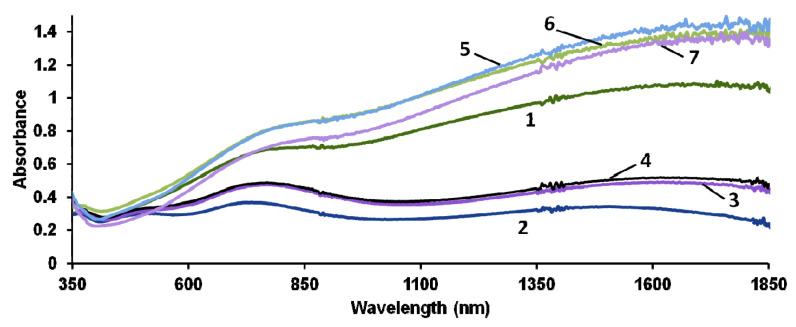
Electronic UV–Vis–NIR absorption spectra of films of PEDOT complexes with PAMPSA (1), t-PASA (2), PAMPSA/t-PASA (1:1) (3), PAMPSA/t-PASA (2:1) (4), PAMPSNa (5), t-PASNa (6) and PAMPSNa/t-PASNa (7) obtained during GS synthesis.

Figure 5 shows UV–Vis–NIR absorption spectra of dry PEDOT–polyelectrolyte films on air. In our opinion, the most adequate analysis of the absorptions observed in the range of 700–1850 nm is presented in [44] based on the results of DFT calculations.

First of all, let us consider the spectrum of PEDOT–t-PASA film (curve 2), where the most pronounced absorption maxima are observed near 745 and 1510 nm. These absorption maxima resemble those found in [44] at 791 nm and 1772 nm and are attributed to low-doped PEDOT in the polaronic state. Shorter wavelengths, at which the polaronic maxima are experimentally observed for PEDOT–t-PASA film, may be explained by the shorter conjugation length or even shorter chain length of the polymer. It is important that one can observe an absorption deep in the range of 900–1350 nm, where the authors of [44] predict absorption maxima near 915, 933 and 1300 nm for the bipolaronic state of PEDOT. Contrarily, in the spectra of PEDOT films prepared in flexible-chain polyacid and salt forms of all polyelectrolytes and their mixtures, one can observe the full range of absorptions predicted for both polaronic and bipolaronic states of PEDOT. The spectra of PEDOT films prepared in the polyacid mixtures (curves 3,4 corresponding to 1:1 and 2:1 compositions) have a similar shape and look more like this one of PEDOT–t-PASA film, but the absorptions near 745 and 1510 nm are shifted to 770 and 1635 nm, respectively. Since in these spectra 3 and 4 one can also observe the absorption deep in the range of 900–1350 nm indicating the low content of the bipolarons, it is reasonable to suppose that the above-mentioned shift is due to longer conjugation.

These peculiarities of the electronic spectra of PEDOT–polyelectrolyte films may be explained on the basis of differences in polyelectrolytes’ structure. The main structural differences between them are: the flexibility of the main polymer chain; the length and flexibility of side chains bearing sulfonic groups; and the distribution of sulfonic groups along the main chain. It is clear from Figure 1 that if one considers a simplified variant of the double-strand interpolymer complex, the rigid-chain t-PASA(Na) with irregular distribution of sulfonic groups with short links to the main chain cannot fully compensate double charges of bipolaron fragments on the rigid conjugated PEDOT chain. This results in obstacles for the elongation of the polymer chain during EDOT electropolymerization in the presence of t-PASA. On the contrary, flexible-chain PAMPSA(Na), thanks to long and flexible side chains, can fully compensate these charges. However, one should admit that the rigid-chain polyelectrolyte may also form interpolymer complexes, in which the polyelectrolyte chains are crossing (almost perpendicularly) several PEDOT chains. So, in the case of EDOT electropolymerization in the presence of polyacid mixtures, PAMPSA can compensate the charges that t-PASA failed to compensate due to steric hindrances (Figure 1). This may contribute to the formation of longer PEDOT chains.

The differences in charge compensation by acid and salt forms of t-PASA were explained by us earlier on the basis of zeta-potential measurements [11]. It was shown that sulfonic groups in t-PASA in the acid form are approximately 3 times less negatively charged (less dissociated) than in the Na-salt form. Another electrostatic obstacle for PEDOT chain charge compensation in the case of rigid-chain t-PASA may arise from hydrogen bond interaction between the sulfonic group and adjacent NH group detected by us using FTIR spectroscopy [11]. This interaction produces positive charges on the polyacid chain, which repulse from positively charged moieties of oxidized forms of PEDOT. This type of interaction was not detected for t-PASNa due to the absence of movable protons in its structure. The above factors may be used to explain cardinal differences in the spectra of PEDOT–t-PASA and PEDOT–t-PASNa films.

#### 3.2.2. Spectroelectrochemical Study of Redox Processes in PEDOT-PE Films

**Figure 6:**
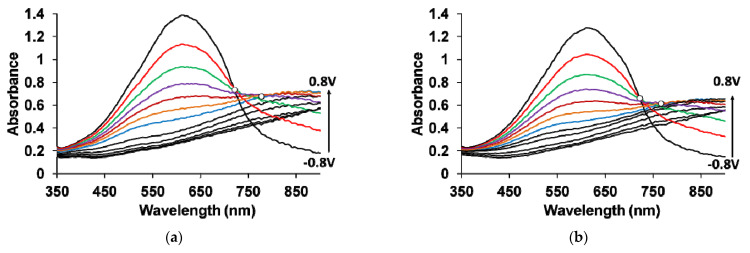

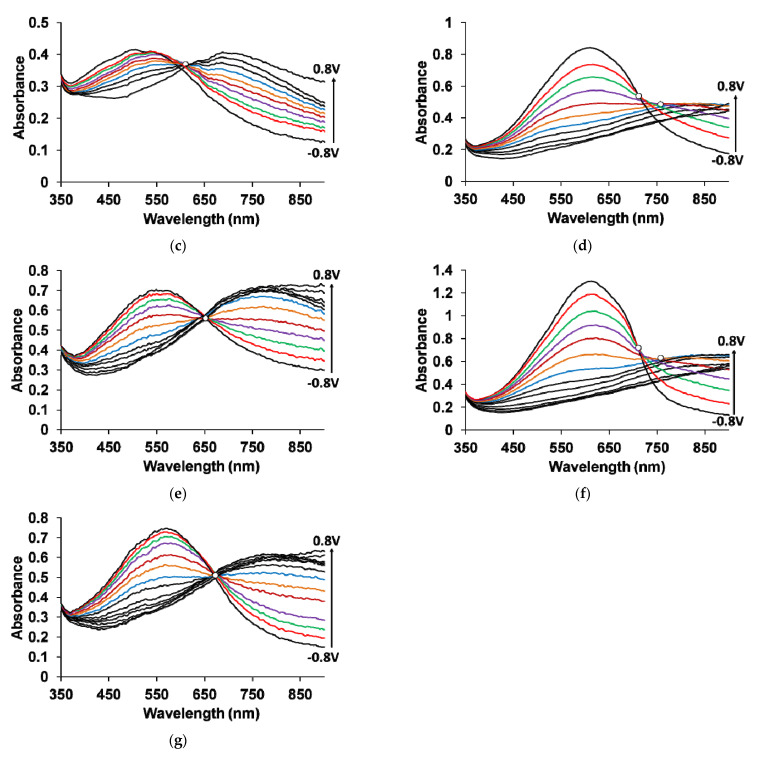
Optical absorption spectra of PEDOT films prepared in PAMPSA (**a**), PAMPSNa (**b**), t-PASA (**c**), t-PASNa (**d**), PAMPSA/t-PASA 1:1 (**e**), PAMPSNa/t-PASNa 1:1 (**f**) and PAMPSA/t-PASA 2:1 (**g**) measured at different potentials in 0.5 M aqueous solution of NaClO_4_.

Figure 6 shows the absorption spectra of PEDOT films at fixed potentials in the aqueous solution of 0.5 M NaClO_4_. A pronounced band near 612 nm due to π–π* transitions in the reduced form of PEDOT (Figure 6a,b,d,f, Table 2) [37,40,41,42,43] can be seen at low potentials in the spectra of PEDOT films obtained in the presence of PAMPSA and salt forms of all PEs and the mixtures. With an increase in the potential (oxidation), the intensity of this band decreases, while at the same time an absorption band at about 800–900 nm is formed (the polaronic form of the oxidized PEDOT). During this transition, an isosbestic point (i.p.1) appears near 710–720 nm. Further growth in the potential causes an increase in absorption in the NIR region of the spectrum (transition from polaronic to bipolaronic form) [40,41,42,43] and the second isosbestic point (i.p.2) can be observed. In reality, the transitions from the neutral form to the polaronic form and then to the bipolaronic form occur in wider ranges of potential, but optical phenomena occurring during PEDOT doping/dedoping due to changes in the film thickness and its refractive index introduce some uncertainty in the isosbestic points’ positions (sometimes called “isosbestic range”) [32].

The charter of changes in the electronic absorption spectra for PEDOT–t-PASA film shows significant differences (Figure 6c) from this one for traditional PEDOT films [30,31,32]. The maximum of the characteristic absorption bands of reduced (~500 nm) and polaron fragments (~700 nm) of PEDOT is shifted hypsochromically compared to that for PEDOT films obtained in the presence of PAMPSA and salt forms of all PEs and the mixtures (Table 2). The observed shift to the short-wave region as discussed above may be due to a decrease in the length of the π-conjugation of the polymer chain and the formation of short PEDOT chains. In addition, the retarded formation of a highly conductive bipolaronic form in this film is observed, as can be seen from the low absorption in the NIR region. PEDOT–t-PASA film has only one isosbestic point shifted to the short-wave region (611 nm).

PEDOT films obtained in the mixtures of polyacids (even at twice higher content of the flexible-chain polyacid) (Figure 6e,g) demonstrate behavior similar to that of PEDOT–t-PASA film: the maximum of the absorption bands of the reduced (~550 nm) and polaronic (~770 nm) forms of PEDOT and the i.p.1 (650–670 nm) are shifted to the short-wave region and are located in the position between those for the films of complexes with rigid-chain and flexible-chain polyacids. It is characteristic that the isosbestic point in this case exists in the whole range of studied potentials, indicating preferential conversion of the neutral form of PEDOT to the polaronic one, and, to a much lesser extent, to the bipolaronic one. Thus, PEDOT–polyacid mixture films demonstrate retarded formation of bipolarons as PEDOT–t-PASA, but have longer conjugation.

So, the rigid-chain polyacid t-PASA produces a dominating influence on the spectroelectrochemical properties of PEDOT–PAMPSA/t-PASA films. Previously, the domination effect of the rigid-chain t-PASA in mixtures with the flexible-chain PAMPSA was first discovered by us during the electrosynthesis of PANI [33]. Now it is clear that this is a universal phenomenon.

#### 3.2.3. Morphology of PEDOT-PE Films

**Figure 7:**
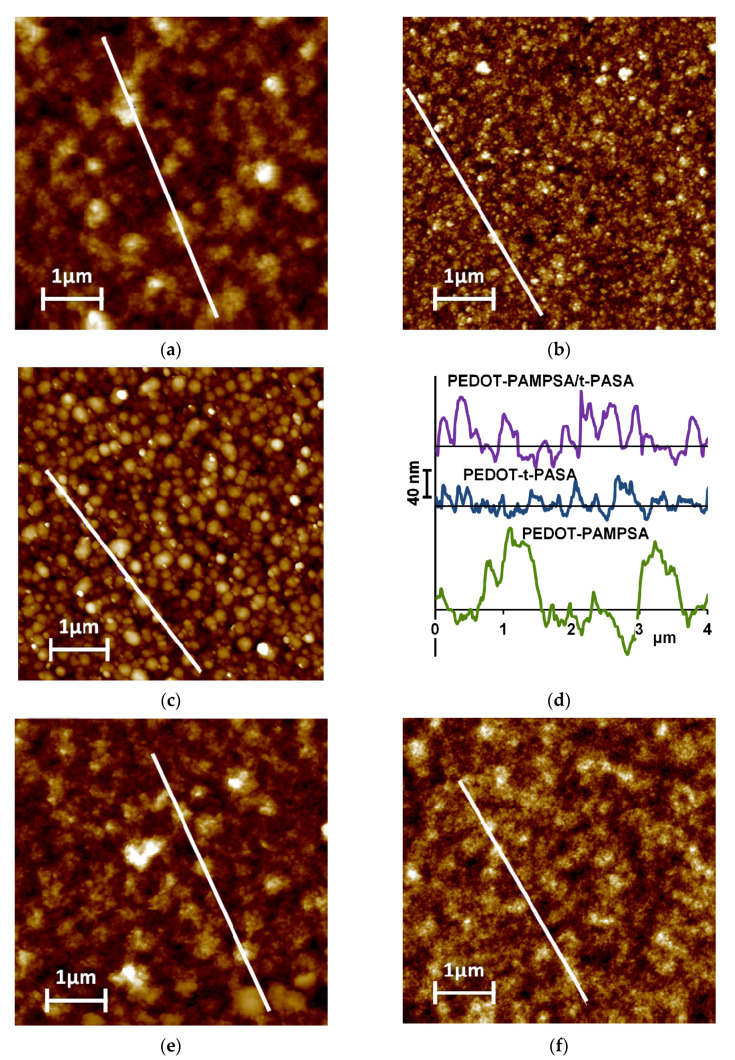

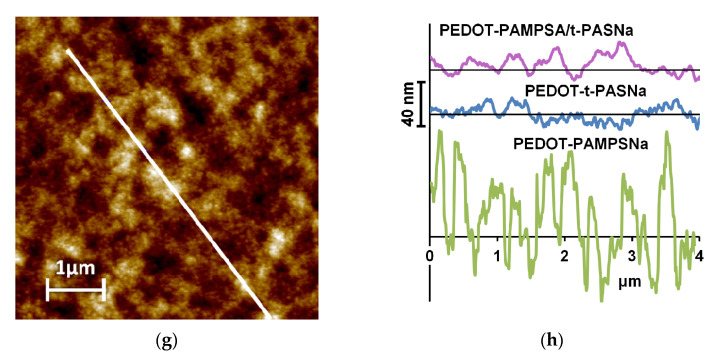
AFM images of PEDOT films obtained in the presence of PAMPSA (**a**), t-PASA (**b**), PAMPSA/t-PASA (1:1) (**c**), their cross-section profiles along white line on the images (**d**), PAMPSNa (**e**), t-PASNa (**f**), PAMPSNa/t-PASNa (**g**) and their cross-section profiles (**h**).

Films of PEDOT, obtained in the presence of PEs, were also characterized using the AFM method. Figure 7 shows that films with greater roughness formed by larger objects are obtained during the synthesis of PEDOT in the presence of PAMPSA, PAMPSNa and, to a lesser extent, in the mixtures of polyacids and polysalts. PEDOT films obtained in the presence of PAMPSA and salt forms of PEs and their mixtures reveal filament-like structures. The surface of the films of PEDOT with t-PASA and 1:1 mixture of the polyacids consists of isolated globules with a size of 150–400 nm.

### 3.3. Ammonia Sensing Properties of PEDOT-PE Films Using Optical Detection Method

**Figure 8:**
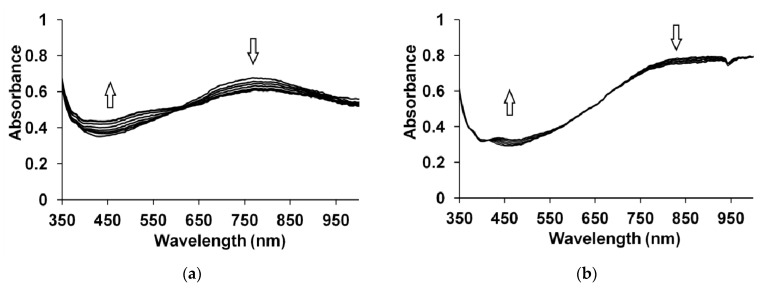
Changes in the optical absorption spectra of (**a**) PEDOT–PAMPSA/t-PASA and (**b**) PEDOT–PAMPSNa/t-PASNa films during exposure to ammonia vapors with the concentration of 25 ppm.

Figure 8 shows the changes in the optical absorption spectra of PEDOT–PAMPSA/t-PASA (1:1) (a) and PEDOT–PAMPSNa/t-PASNa (b) films exposed to 25 ppm NH_3_. Changes in the spectra shown in Figure 8a are specific for PEDOT films obtained in the presence of t-PASA and polyacid mixture. Changes in the spectra shown in Figure 8b are typical for PEDOT films obtained in PAMPSA and all salt forms of PEs.

The influence of ammonia on the PEDOT films leads to their reduction [30,31,32], as evidenced by an increase in the absorption in the region of the reduced form (450–500 nm) and a decrease in the absorption in the region of polarons (800–850 nm).

**Figure 9:**
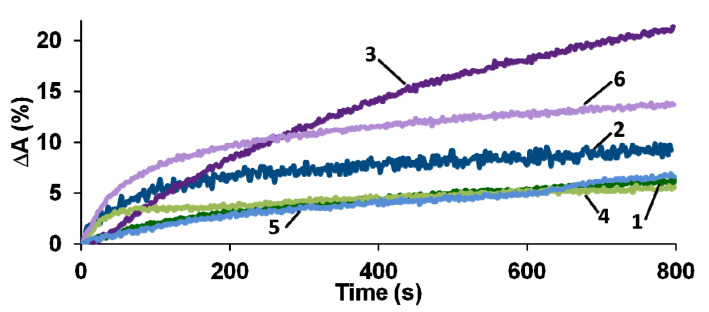
The response transients for PEDOT films obtained in the presence of PAMPSA (1), t-PASA (2), PAMPSA/t-PASA (1:1) (3), PAMPSNa (4), t-PASNa (5) and PAMPSNa/t-PASNa (6) exposed to 25 ppm NH_3_.

For the PEDOT films obtained in the presence of PAMPSA, salt forms of PEs and their mixtures, much smaller changes in the absorption are observed than those in the case of PEDOT films with the rigid-chain t-PASA and the PAMPSA/t-PASA mixture (Figure 8a). In most cases, a greater change in the absorption is observed in the region of 450–500 nm. Hence, the values of amplitude response ∆A were calculated at this wavelength region and their time dependences are presented in Figure 9. Subsequently, a blue or blue-green LED, emitting in the range (450 < λ < 500 nm), can be used in a commercial device to detect changes in absorption in this wavelength range. All calculated values of the sensor responses for all investigated films are presented in Table 3.

It can be seen from Table 3 that PEDOT–t-PASA and PEDOT–PAMPSA/t-PASA films are characterized by higher ∆A and shorter response times than PEDOT–PAMPSA, the PEDOT mixed film demonstrating the highest response. Even at a very low concentration of ammonia 5 ppm (less than the MPC), the mixed film is capable of detecting ammonia with sufficient efficiency and accuracy. PEDOT films obtained in the salt forms of PEs showed the same tendency but their ∆A are several times lower. However, they reveal significantly shorter response times. The latter fact may be explained by the presence of excessive protons in the film, which partly neutralize ammonia penetrating to the surface [32].

The observed difference in sensing properties may be explained on the basis of spectroelectrochemical data. Upon reduction (Figure 6c,e) or exposure to ammonia, the PEDOT films obtained in t-PASA and PAMPSA/t-PASA mixture preferentially exhibit a transition from the polaronic form to the reduced one. So, PEDOT interaction with ammonia results in only one direct electronic transition. The surface morphology of PEDOT films can also affect their sensing properties. The surfaces of the PEDOT films obtained in t-PASA and a mixture of polyacids consist of isolated globules, which probably allow ammonia to interact with a larger surface area of the film. For sensors based on PANI, a similar correlation between globular morphology and sensing properties was found [45,46]. On the other hand, the roughness of PEDOT–t-PASA is the lowest, while higher roughness should increase the contact area of the film [46]. So, in the case of the mixed PEDOT–polyacid film, the reduced number of electronic transitions and globular morphology due to the presence of t-PASA and higher roughness due to the presence of PAMPSA contribute to the best sensor performance.

## 4. Conclusions

As a result of the present study, it was found that PEDOT films can be electrodeposited in aqueous solutions of mixtures of polyelectrolytes distinguished by flexibility of the polymer chain. The electrosynthesis of PEDOT films in the presence of the salt forms of the mixed polyelectrolytes proceeds in a similar manner as that for PEDOT electropolymerization in aqueous solutions of flexible chain PEs or inorganic electrolytes. The resulting PEDOT films have a similar electronic structure, surface morphology and electrochemical and spectroelectrochemical properties. So, in this case, the presence of rigid-chain PEs in the PAMPSNa/t-PASNa mixture does not affect the character of PEDOT electrosynthesis and the properties of the films obtained.

On the contrary, if one uses acid forms of the rigid- and flexible-chain polyelectrolytes in the mixture, the evolution of electronic spectra during PEDOT electrosynthesis has an intermediate character between those in the presence of flexible- and rigid-chain polyacids, the electropolymerization rate being higher and closer to that in the latter case. This higher electropolymerization rate may be explained by the fact that thanks to the presence of the rigid-chain polyacid in the PAMPSA/t-PASA mixture the intermediate EDOT oligomers have a higher content of polaronic form, being the main driving force for PEDOT chain growth.

Surprisingly, the electronic absorption spectra, spectroelectrochemical properties and surface morphology of the electrodeposited PEDOT–PAMPSA/t-PASA films are almost identical to those of PEDOT films obtained in the presence of the rigid-chain polyacid. The evolution of in situ absorption spectra with potential indicates the retarded formation of the bipolaronic form of PEDOT in this case. So, the presence of a rigid-chain polyacid in PAMPSA/t-PASA mixtures produces a decisive influence on the electronic structure of the electrodeposited PEDOT films even at twice higher content of flexible-chain polyacid. In this respect, the mixed PEDOT films resemble those of polyaniline studied in our earlier publications [33].

The electrodeposited PEDOT films with the individual PEs and their mixture were comparatively tested as optical ammonia sensors. It was shown that all PEDOT films can reliably detect 25 ppm ammonia, with the mixed PEDOT–PAMPSA/t-PASA films demonstrating the best sensor performance due to the synergistic effect of two factors: (1) upon exposure to ammonia, the presence of rigid-chain polyacid provides rapid polaron–neutral transition and (2) the flexible-chain polyacid gives high roughness and contributes to the easier penetration of ammonia into the film. It is important to note that, similarly to all the sensors based on popular conducting polymers (polyaniline, polythiophene, polypyrrole and their derivatives), the PEDOT films described in this study are sensitive to all oxidative or reductive gases and vapors. However, studying this interference was not among the aims of this work.

## Figures and Tables

**Table 1:** Electrochemical parameters of different regimes of PEDOT electrosynthesis in the presence of various PEs and their mixtures.

PEs	E_PC_, V	T_PS_, s	I_PS_, mA	E_GS_, V
PAMPSA	0.86	8	0.18	0.87
t-PASA	0.84	8	0.47	0.88
PAMPSA/t-PASA (1:1)	0.80	8	0.39	0.87
PAMPSA/t-PASA (2:1)	0.81	8	0.40	0.89
PAMPSNa	0.88	10	0.14	0.87
t-PASNa	0.89	25	0.26	0.87
PAMPSNa/t-PASNa (1:1)	0.88	18	0.17	0.87

**Table 2:** The positions of the maxima of the absorption bands of the reduced and polaronic forms of PEDOT and the isosbestic points i.p.1 (neutral-to-polaron) and i.p.2 (polaron-to-bipolaron).

PEs	Reduced Form, nm	i.p.1, nm (Potential Range *, V)	Polaronic Form, nm	i.p.2, nm (Potential Range *, V)
PAMPSA	612	722 (−0.8 ÷ −0.6)	835	777 (−0.5 ÷ −0.2)
t-PASA	505	611 (−0.6 ÷ 0.2)	690	-
PAMPSA/t-PASA (1:1)	548	653 (−0.6 ÷ 0.3)	774	-
PAMPSA/t-PASA (2:1)	570	674 (−0.7 ÷ 0.3)	775	-
PAMPSNa	612	727 (−0.8 ÷ −0.5)	832	772 (−0.5 ÷ −0.3)
t-PASNa	611	728 (−0.6 ÷ −0.4)	828	754 (−0.4 ÷ −0.2)
PAMPSNa/t-PASNa (1:1)	613	714 (−0.8 ÷ −0.5)	845	756 (−0.4 ÷ −0.2)

Note: * the ranges of potential, in which the polaronic and bipolaronic forms exist, in reality are wider (see the discussion).

**Table 3:** Values of response amplitude (∆A) and response time (t_r_) of PEDOT films.

PEs	∆A at 25 ppm, %	∆A at 5 ppm, %	t_r_, s (25 ppm)
PAMPSA	6.05	3.17	637
t-PASA	10.19	5.06	616
PAMPSA/t-PASA	20.91	7.26	648
PAMPSNa	5.46	2.17	346
t-PASNa	3.41	1.15	125
PAMPSNa/t-PASNa	13.54	4.21	324

## Data Availability

The data presented in this study are available on request from the corresponding author.
